# Prolonged Fever in a Pediatric Patient: A Case of Systemic Juvenile Idiopathic Arthritis (sJIA) Complicated by Macrophage Activation Syndrome (MAS)

**DOI:** 10.7759/cureus.46083

**Published:** 2023-09-27

**Authors:** Marianne Cortes, Brian G Nudelman, Megan J Rouse, Maria D Frost

**Affiliations:** 1 Osteopathic Medicine, Nova Southeastern University Dr. Kiran C. Patel College of Osteopathic Medicine, Davie, USA; 2 Internal Medicine, Memorial Healthcare System, Pembroke Pines, USA; 3 Pediatrics, Broward Health Medical Center, Fort Lauderdale, USA

**Keywords:** ferritin elevation, prolonged fever, inflammatory polyarthritis, pediatric rheumatology, systemic juvenile idiopathic arthritis, macrophage activation syndrome (mas), hemophagocytic lymphohistiocytosis (hlh)

## Abstract

A prolonged fever in a child can be due to a range of causes including infectious, autoimmune, malignant, or genetic in etiology. In our report, we present the case of a previously healthy three-year-old female diagnosed with macrophage activation syndrome (MAS) due to complications of systemic juvenile arthritis (sJIA). MAS is considered a secondary subtype of hemophagocytic lymphohistiocytosis (HLH), a rare and life-threatening group of syndromes characterized by overstimulation of the immune system leading to systemic inflammation. Through our case, we wanted to bring awareness to this uncommon group of diseases as well as discuss the importance of differentiating between its subtypes. While HLH and MAS have similar clinical presentations, the treatment regimen for each is distinct. Moreover, further research should be conducted to create standardized criteria and treatment guidelines that are evidence-based in order to properly manage these patients.

## Introduction

Hemophagocytic lymphohistiocytosis (HLH) is a rare disorder of early childhood characterized by severe systemic hyperinflammation. HLH can be life-threatening due to rapid progression to multi-organ failure and central nervous system involvement, making diagnosis crucial to prevent long-term sequelae [1]. The incidence of HLH has been estimated to be 1-6/300,000 [2]. Etiologies may include genetic mutation, infection, malignancy, and rheumatologic disorders [1,3]. The rheumatologic equivalent of HLH is macrophage activation syndrome (MAS), which occurs as a complication of widespread inflammation seen in conditions like systemic juvenile arthritis (sJIA) [3,4]. MAS is an uncommon complication of sJIA, only seen in 5-8% of cases [4]. Symptomatology mirrors that of malignancy, Multi-system inflammatory syndrome in children (MIS-C), Kawasaki disease (KD), and the non-specific clinical presentation poses diagnostic challenges. In addition to this, the infrequent nature of both of these diseases has led to a lack of evidence-based criteria, making timely management difficult.

This article was previously presented in the 2023 KPCOM Office of Graduate Medical Education (OGME) Scientific Research Poster Competition and 2023 Broward Graduate Medical Education (GME) Symposium.

## Case presentation

We present a case of a previously healthy three-year-old female who presented with four days of fever secondary to respiratory syncytial virus and was admitted for dehydration and sepsis to rule out. Fevers persisted for over a week ranging from 101.5-102.4°F with negative blood cultures, significant leukocytosis, and elevated inflammatory markers. Treatment of MIS-C versus KD was pursued. Following two courses of steroids and intravenous immunoglobulin (IVIG), the patient remained febrile and developed acute symptomatic anemia with labs significant for elevated lactate dehydrogenase (LDH), haptoglobin, and reticulocyte count (Table 1). At this time, there was concern for transient erythroblastopenia of childhood (TEC). After three weeks, the patient remained afebrile for 24 hours and was discharged. Upon evaluation in the outpatient setting, the mother reported the return and persistence of fevers, a faint pink macular rash with excoriations on the trunk and flank, and an inability to bear weight. The patient was referred for admission for oncologic workup and subsequently underwent bone marrow biopsy, pan CT, MRI, and PET scans which were only significant for reactive lymphadenopathy. There was concern for HLH versus sJIA with MAS at this time due to prolonged fevers with uptrending ferritin levels (>6000), elevated CD25, and polyarthritis. The patient began treatment with IVIG, steroids, IL-1, and IL-6 inhibitors due to increased suspicion of sJIA due to rash and polyarthritis. The patient was initially refractory to IL-2 inhibitor but later showed an appropriate response to IL-6 inhibitor with significant clinical improvement and downtrending ferritin, which supported diagnosis for sJIA complicated by MAS. Once the patient was stabilized, she was discharged and referred to a rheumatologist for continuous follow-up. She has significantly improved since discharge, depicted by a drop in the ferritin level and resolution of symptoms in response to IL-6 and methotrexate. She continues biologic therapy in an outpatient setting. 

**Table 1:** Patient's laboratory findings. CRP: C-reactive protein; ESR: erythrocyte sedimentation rate; ALT: alanine transaminase; AST: aspartate aminotransferase

Lab	Value	Normal Findings
WBC	36.1 x 10^9^/L	4.5-11 x 10^9^/L
Platelets	564 x 10^9^/L	140-400 x 10^9^/L
ESR	129 mm/h	<20 mm/hr
CRP	18.3 mg/L	8-10 mg/L
ALT	72 IU/L	10-40 IU/L
AST	23 IU/L	10-34 IU/L
Ferritin	9885 ng/mL	7-142 ng/mL
Fibrinogen	596 mg/dL	200-400 mg/dL
Total protein	8.8 g/dL	6-8.3 g/dL

## Discussion

This case highlights the clinical presentation and progression of a pediatric patient with MAS, a subtype of secondary HLH. The most common etiologies of MAS are sJIA in children and Still’s disease in adults [2,5]. It may be difficult to differentiate sJIA complicated by MAS versus sJIA alone due to an overlap of symptoms between the two. Characteristics that help differentiate sJIA complicated by MAS include the absence of arthritis and serositis along with persistent fever (Table 2).

**Table 2:** Differentiating the symptoms and lab values of systemic juvenile idiopathic arthritis (sJIA) versus that of sJIA complicated by macrophage activation syndrome (MAS). Table created from "Symptoms of sJIA and MAS in association with sJIA" by Avau and Matthy. Used under Creative Commons 4.0 International license. Link: https://www.researchgate.net/publication/284770995_Therapeutic_Potential_of_Interferon-g_and_Its_Antagonists_in_Autoinflammation_Lessons_from_Murine_Models_of_Systemic_Juvenile_Idiopathic_Arthritis_and_Macrophage_Activation_Syndrome CRP: C-reactive protein; ESR: erythrocyte sedimentation rate; ALT: alanine transaminase; AST: aspartate aminotransferase

	sJIA	sJIA complicated by MAS
Fever	Quotidian	Persistent
Rash	Evanescent	Petechial/macular
Hepato/Splenomegaly	+	+
Lymphadenopathy	+	+
Arthritis	+	-
Serositis	+	-
Neutrophil count	↑↑	↓
Platelet count	↑↑	↓
ESR	↑↑	Normal or ↓
CRP	↑	↑
ALT/AST	Normal or ↑	↑↑
Ferritin	Normal or ↑	↑↑
D-dimer	↑	↑↑

There is an increased risk of MAS in patients with a previous history of autoimmune disorders, infections, and periodic fever syndromes [1,2,5]. Previous literature has seen infection by Epstein-Barr virus as an especially common trigger for both primary and secondary HLH, although not seen in our case [1]. The pathogenesis of MAS is thought to be caused by an overwhelming immunologic response to a specific trigger, leading to continuous activation and expansion of T lymphocytes and macrophages which results in massive hypersecretion of proinflammatory cytokines [5]. In our case, we know this trigger to be autoinflammation caused by sJIA.

Once HLH is suspected, bone marrow, cerebrospinal fluid, genes, and markers of immune system activation (ferritin, IL-2, CD25) should be evaluated [1-3,5]. In addition to these markers of inflammation, sJIA diagnostic criteria include intermittent fevers, arthritis for at least two weeks, and one of the following: rash, general lymphadenopathy, hepatosplenomegaly, or serositis [1]. While central nervous system (CNS) involvement is not included in the criteria, it is important to note that it occurs in 30% to 73% of HLH patients in the form of seizures, focal deficits, meningismus, and altered levels of consciousness [3]. The involvement of CNS symptomatology has been correlated with worse clinical outcomes and possible permanent neurologic sequelae [1,3]. Polyarthritis, inability to bear weight, and rash were factors that directed treatment toward sJIA with MAS in our case, which includes steroids and biologics [4]. This treatment plan differs from the traditional treatment course of HLH which includes steroids and chemotherapy (i.e etoposide) [1-3]. Both treatment modalities ultimately suppress inflammatory response secondary to cytokine storming through different mechanisms. Differences in approaches to treatment make the distinction of HLH subtypes so crucial. In addition to this, supportive care should also be taken in mind when treating patients with these conditions to minimize pain such as adequate nutrition, calcium, and vitamin D supplements [4].

## Conclusions

Pediatric HLH continues to challenge physicians with its indistinct course of symptoms and lack of availability of efficient and specific testing to minimize deadly delays in diagnosis. In our case, we demonstrated the importance of differentiating traditional HLH from that of a secondary subtype of HLH, MAS. The specific symptoms of polyarthritis, inability to bear weight, and rash were factors that pushed towards the treatment of MAS instead of HLH, leading to the initiation of the appropriate life-saving treatment for our patient. Due to the high mortality rate associated with HLH and its subtypes, it is imperative that physicians consider HLH in the differential for patients presenting with a prolonged, non-specific clinical course with evidence of widespread systemic inflammatory response.
